# Multi-omics landscape and functional validation of HCCS in breast cancer: from pan-cancer immunometabolic characterization to regulating tumor proliferation

**DOI:** 10.3389/fgene.2026.1777429

**Published:** 2026-05-19

**Authors:** Hao Cheng, Chaolin Li

**Affiliations:** 1 Department of Breast Surgery, Jinniu Maternity and Child Health Hospital of Chengdu, Chengdu, Sichuan, China; 2 Department of Academic Affairs, Jinniu Maternity and Child Health Hospital of Chengdu, Chengdu, Sichuan, China

**Keywords:** biomarkers, drug sensitivity, HCCS, immunotherapy, pan-cancer, prognosis

## Abstract

**Background:**

Holocytochrome c synthase (HCCS) is an essential mitochondrial regulator; however, its pan-cancer significance and its direct functional role in breast cancer (BRCA) progression remain insufficiently explored. This study aimed to systematically characterize the pan-cancer landscape of HCCS and validate its biological impact on BRCA.

**Methods:**

We integrated multi-omics data from 33 TCGA cancer types to investigate HCCS expression patterns, genomic alterations, and its correlation with the immune microenvironment. In breast cancer cell lines (MDA-MB-231), the effects of HCCS knockdown on cell proliferation and cell cycle distribution were assessed using CCK-8, colony formation, and flow cytometry assays. Finally, we constructed a machine learning-based prognostic signature and performed drug sensitivity analysis to evaluate its clinical relevance.

**Results:**

HCCS was significantly upregulated in multiple malignancies, including BRCA, and correlated with poor patient prognosis. Pan-cancer analysis suggested that HCCS might act as a mitochondrial-immunometabolic hub involved in immune evasion. *In vitro* validation confirmed that HCCS is a key driver of tumor growth, as HCCS knockdown significantly inhibited cell proliferation and colony-forming ability by inducing G0/G1 phase arrest. By integrating these mechanistic findings with clinical data, we developed a robust HCCS-based prognostic model that demonstrated high predictive accuracy across independent BRCA cohorts. Furthermore, pharmacogenomic analysis identified candidate small molecules, such as MK-886 and mercaptopurine, as potential therapeutic options.

**Conclusion:**

Our findings transition HCCS from a computational biomarker to a functionally validated regulator of breast cancer cell survival. By linking its systemic immunometabolic impact with its direct role in cell cycle control, this study identifies HCCS as a promising therapeutic target and a reliable prognostic indicator in BRCA.

## 1 Introduction

Malignancies constitute the second leading cause of death globally (Bray et al., 2024). The clinical management of cancer is significantly impeded by high heterogeneity and the development of resistance to immunotherapy (Wu et al., 2021). While established biomarkers like TMB and PD-L1 expression are widely used, their predictive power is often limited by tumor-specific metabolic contexts (Yang et al., 2023; Sun et al., 2023; Mastrolonardo et al., 2025). The intricate interplay between metabolic reprogramming and the tumor immune microenvironment (TME) necessitates a functional pan-cancer characterization of core mitochondrial molecules (Tharp et al., 2024). As a central regulator of the electron transport chain, HCCS may represent a functional nexus where metabolic state dictates immune cell fitness, offering a more biology-driven alternative to current universal biomarkers.

Recent advances have underscored that mitochondrial metabolic reprogramming is not merely a bystander in oncogenesis but a pivotal driver that reshapes the tumor-immune microenvironment (Kuo et al., 2022). By modulating the availability of key metabolites and signaling molecules, dysfunctional mitochondria can impair the anti-tumor activity of infiltrating immune cells, thereby promoting immune evasion and therapeutic resistance (Ahn et al., 2024; Guan et al., 2024). Holocytochrome c synthase (HCCS) is a central regulator of mitochondrial energy metabolism and apoptosis. It maintains the function of the electron transport chain by catalyzing the covalent attachment of heme to cytochrome c (Babb et al., 2014; Babbitt et al., 2014). Previous research has primarily focused on its enzymatic mechanisms—such as the structural functions of the heme-binding domain (Vergult et al., 2013; San Francisco et al., 2013)—and developmental disorders arising from gene mutations, such as microphthalmia syndrome (Wimplinger et al., 2007; Wimplinger et al., 2006; Prakash et al., 2002). However, a significant gap exists in our systematic understanding of HCCS in oncology. Current evidence is largely confined to its oncogenic role in specific lung cancer models (Luo et al., 2025). Beyond respiratory malignancies, preliminary studies have hinted that it may be significantly associated with poor clinical prognosis in breast cancer (Bellah et al., 2025), but these observations remain fragmented. There is a lack of broad cross-cancer profiling and functional validation. Furthermore, while mechanistic studies have linked HCCS to apoptotic dysregulation (Indrieri et al., 2013), its potential association with cell cycle control and TME modulation remains overlooked. Crucially, the clinical relevance of HCCS regarding immunotherapy response and targeted drug resistance has not been explored. These characteristics underscore the potential of HCCS as a novel pan-cancer biomarker, warranting a comprehensive investigation into its clinical utility through integrated multi-omics analysis.

In this study, we present the first multidimensional functional map of the HCCS gene across the pan-cancer landscape. By integrating genomic (CNV/methylation), transcriptomic, proteomic, and spatial transcriptomic data from 33 TCGA cancer types, we systematically analyzed the drivers of HCCS dysregulation. Leveraging platforms such as TIMER 2.0 and TISCH2, we elucidated how HCCS mediates tumor-immune interactions *via* the MDK-LRP1/VEGFA-VEGFR2 signaling axes. We further established clinical associations between HCCS expression and sensitivity to ICIs, as well as resistance to EGFR-targeted therapies, and constructed a consensus prognostic model for breast cancer using machine learning. These findings reposition HCCS as a mitochondrial-immunometabolic hub and provide a theoretical foundation for developing novel HCCS-targeted combination therapies.

## 2 Materials and methods

### 2.1 Dataset acquisition and processing

Data acquisition followed established protocols (Hoadley et al., 2018; Liu et al., 2020). We utilized standardized, normalized, batch-corrected, and platform-corrected RNA matrix files (EBPlusPlusAdjustPANCAN_IlluminaHiSeq_RNASeqV2.geneExp.tsv) generated by the PanCancer Atlas Consortium, comprising 11,069 samples. Curated clinical phenotype data were obtained from the UCSC Xena database, including initial enrollment data and follow-up records for 11,160 patients across 33 cancer types (Liu et al., 2018). Additionally, normal tissue data from GTEx were integrated with TCGA samples *via* the UCSC Xena TOIL hub, which re-processed both datasets using a unified pipeline to minimize batch effects. To further ensure comparability, the Z-score transformation was applied to normalize expression levels, with outliers (|Z| > 3) excluded to enhance data robustness. Pan-cancer transcriptome profiles, copy number variation (CNV) data, and 450K methylation profiles for HCCS were acquired from the same repository. Reverse-phase protein array (RPPA) data were sourced from The Cancer Proteome Atlas (TCPA) (http://www.tcpaportal.org). Immune cell infiltration data were downloaded from Tumor Immune Estimation Resource 2.0 (TIMER2.0) (Li et al., 2020). Single-cell RNA sequencing (scRNA-seq) data were obtained from Tumor Immune Single-cell Hub 2 (TISCH2) (Han et al., 2023). Metrics for the Cancer-Immunity Cycle were derived from the Tracking Tumor Immunophenotype (TIP) database (Xu et al., 2018), and Immunophenoscore (IPS) were acquired from The Cancer Immunome Atlas (TCIA) (Charoentong et al., 2017). Spatial transcriptomic data for breast cancer (BRCA; GSE210616-GSM6433589), pancreatic adenocarcinoma (PAAD; GSE211895-GSM6505134), and skin cutaneous melanoma (SKCM; GSE179572-GSM5420750) were retrieved from the Gene Expression Omnibus (GEO) (Bassiouni et al., 2023; Barkley et al., 2022; Denisenko et al., 2024).

### 2.2 Assessing the pan-cancer expression landscape of HCCS

HCCS expression levels and subcellular localization in normal tissues, immune cells, and tumor cell lines were evaluated using the Human Protein Atlas (HPA). RNA matrices were converted to unitless Z-scores per tumor type. To ensure analytical accuracy, differential expression analysis paired the Cancer Genome Atlas (TCGA) primary tumor tissues with Genotype-Tissue Expression (GTEx) normal tissue data. Pan-cancer HCCS protein expression was assessed using the UALCAN platform, validated by immunohistochemistry (IHC) data from HPA.

Spatial transcriptomic profiling across malignancies was conducted using the Sparkle database (https://grswsci.top/) and SpatialTME platform (https://www.spatialtme.yelab.site/). Tumor microenvironment (TME) cellular composition was deconvolved using the Cottrazm package within SpatialTME (Xun et al., 2023; Shi et al., 2024). Sparkle integrated 10x Visium sequencing data from SpatialTME to construct comprehensive spatial transcriptome maps. Raw gene expression counts were normalized using the NormalizeData function. Spatial distributions of predominant cell types were visualized *via* Seurat’s (v4.3.0) SpatialPlot function. Spearman correlation analysis quantified: 1) intercellular abundance correlations across all tissue spots, and 2) associations between cellular composition and gene expression, visualized using linkET. To examine spatial heterogeneity, microregions were stratified by malignant cell proportion: >0% malignant cells (malignant, Mal) versus 0% malignant cells (non-malignant, nMal). Cross-cancer single-cell transcriptomes from Tumor Immune Single-cell Hub 2 (TISCH2) were integrated to generate cell-subset-specific HCCS expression heatmaps using pheatmap.

### 2.3 Genomic alterations of HCCS in pan-cancer

The cBioPortal platform (http://www.cbioportal.org/) assessed pan-cancer frequencies of HCCS mutations, amplifications, and deletions. Using downloaded copy number variation (CNV) data, we evaluated CNV profiles and calculated Spearman correlations with HCCS mRNA expression. Prognostic differences between wild-type and mutant HCCS were examined. HCCS genomic status associations with immune response were analyzed using the immunogenomic framework established by Thorsson et al. (Thorsson et al., 2019). Differential methylation of HCCS probes in tumor *versus* normal tissues was evaluated using 450K methylation data, with Spearman correlation determining HCCS methylation-mRNA expression relationships. Clinically relevant alternative splicing (AS) events were identified *via* the Clinical Alternative Splicing (ClinicalAS) tool in OncoSplicing (http://www.oncosplicing.com/) (Zhang et al., 2025), with validation in SpliceSeq and SplAdder databases. Percent Spliced In (PSI) values from TCGA malignancies and GTEx normal tissues were visualized using PanPlot, comparing tumor-normal PSI differences for cancer-specific AS events.

### 2.4 Functional enrichment analysis

The GeneMANIA database (http://genemania.org/) was used to construct the HCCS protein-protein interaction network. Using the comPPI database (https://comppi.linkgroup.hu/) (Veres et al., 2015), we filtered interacting proteins lacking shared subcellular localization with HCCS. Tumor samples were stratified into high- and low-HCCS expression groups based on thresholds ±30% relative to median expression. Differential expression analysis performed with the limma package (v3.58.1) yielded log_2_ fold-change (log_2_FC) values for all genes. Genes ranked by absolute log_2_FC underwent Gene Set Enrichment Analysis (GSEA) using clusterProfiler (v4.10.0) with Hallmark and Kyoto Encyclopedia of Genes and Genomes (KEGG) metabolic gene sets. The analysis parameters were set as follows: permutations = 1000, gene set size limits = 10–500, and p-value cutoff = 0.05. The normalized enrichment score (NES) were calculated, and multiple hypothesis testing was performed using the Benjamini–Hochberg (BH) method to obtain adjusted p-values. Spearman correlations between HCCS expression and functional proteins from The Cancer Proteome Atlas (TCPA) were computed per sample using cor.test. The top five positively and negatively correlated proteins were selected for visualization. Activity scores for ten cancer-related pathways (TSC/mTOR, receptor tyrosine kinase [RTK], RAS-MAPK, PI3K-AKT, estrogen receptor [ER], androgen receptor [AR], epithelial-mesenchymal transition [EMT], DNA damage response, cell cycle, and apoptosis) were calculated as described previously (Liu et al., 2023). Patients were dichotomized by median HCCS expression, and pathway activities were compared between groups. Functional state z-scores for 14 tumor cell states (defined by the Cancer Single-cell State Atlas [CancerSEA]) were computed using the zscore parameter in Gene Set Variation Analysis (GSVA) (Yuan et al., 2019). These scores were scaled to generate gene set scores. Pearson correlations between HCCS expression and each gene set score were assessed. Finally, enrichment scores for immune-inflammation pathways derived from KEGG gene sets were calculated *via* single-sample GSEA (ssGSEA) and correlated with HCCS expression.

### 2.5 Pan-cancer immunological signatures associated with HCCS

The ESTIMATE algorithm evaluated immune and stromal scores in tumors, with Pearson correlations calculated against HCCS expression. Using cor.test, we analyzed pan-cancer associations between HCCS and both immune regulatory factors and immune checkpoint genes. Correlations between HCCS and Cancer-Immunity Cycle activity were further assessed. Multiple deconvolution algorithms computed Spearman correlations between HCCS expression and immune cell subsets, with results visualized in pan-cancer heatmaps. For scRNA-seq datasets, Uniform Manifold Approximation and Projection (UMAP) dimensionality reduction visualized gene expression patterns. Cell subpopulations were classified as HCCS-positive or HCCS-negative based on expression thresholds. Subpopulation proportions were quantified to identify primary cellular sources of HCCS expression. Intercellular communication networks were inferred using CellChat (Jin et al., 2021).

The Estimation of Systems Immune Response (EaSIeR) tool leverages cancer-specific models to predict antitumor immune responses and biomarkers from RNA sequencing (RNA-seq) data (Lapuente-Santana et al., 2021). Its biomarkers were experimentally validated (Kong et al., 2022), with predictive performance confirmed in four independent anti-PD-1/PD-L1 therapy cohorts. The TCIA provided pan-cancer IPS data predictive of response to anti-CTLA-4/PD-1 inhibitors. Differences in IPS between high- and low-HCCS expression groups were evaluated. The Tumor Immune Dysfunction and Exclusion (TIDE) platform predicts transcriptomic immunotherapy biomarkers using pretreatment tumor profiles (Jiang et al., 2018). Through TIDE’s Biomarker Evaluation module, we compared HCCS with established biomarkers for therapeutic efficacy and overall survival prediction. Cytokine treatment effects on HCCS expression were assessed using the Tumor Immune Syngeneic MOuse tool (TISMO; http://tismo.cistrome.org/) (Zeng et al., 2022). Finally, Kaplan-Meier Plotter (https://kmplot.com/analysis/) evaluated correlations between HCCS expression and immunotherapeutic outcomes.

### 2.6 Association between HCCS and pan-cancer clinical diagnosis and prognosis

Receiver operating characteristic (ROC) curve analysis was performed using the pROC package in R. We calculated the area under the curve (AUC) with 95% confidence intervals (CI) and generated smoothed ROC curves to evaluate HCCS diagnostic performance in discriminating tumor from normal tissues. Univariate Cox proportional hazards regression (survival package) assessed the impact of HCCS expression on clinical prognosis across cancer types, reporting hazard ratios (HR) with 95% CIs. Survival analysis employed the Kaplan-Meier method, with optimal cutoffs for high/low HCCS expression groups determined using the survminer package (v0.4.9). To minimize class imbalance artifacts, a minimum group proportion threshold of 0.3 was enforced. This extreme-group stratification approach, aimed at enhancing the contrast between biological phenotypes and minimizing the influence of intermediate expression levels, has been previously validated in similar transcriptomic studies (Xu et al., 2025). Intergroup survival differences were assessed *via* log-rank tests (survfit function). Restricted cubic splines (RCS) modeled nonlinear relationships between HCCS expression and four survival endpoints: overall survival (OS), disease-specific survival (DSS), progression-free interval (PFI), and disease-free interval (DFI). Both univariate and multivariate Cox analyses evaluated HCCS alongside traditional clinical variables. Forest plots generated with the forestplot package visualized effect sizes and confidence intervals.

### 2.7 Screening of HCCS-related genes and construction of a consensus prognostic model using machine learning

In BRCA samples, HCCS expression levels were stratified into high/low groups based on upper/lower tertiles (30% quantiles). Differential expression analysis (limma package) identified significantly differentially expressed genes (DEGs; |log_2_FC| > 0.585 [1.5-fold change], adjusted p < 0.05), visualized *via* volcano plots. Weighted Gene Co-expression Network Analysis (WGCNA) identified modules co-varying with HCCS expression. The TCGA-BRCA cohort, including transcriptomic profiles and corresponding clinical follow-up data, was utilized as the discovery (training) dataset. To ensure the generalizability of our HCCS-based signature, multiple independent datasets from the GEO databases were curated as external validation cohorts. Specifically, all expression data were log2-transformed and normalized *via* z-score scaling to minimize batch effects. Following established methodology (Liu et al., 2022), we constructed prognostic models using multiple algorithms: LASSO regression, elastic net, ridge regression, stepwise Cox regression, and CoxBoost. Risk scores were computed by linear combination of gene expression values and model coefficients. Model performance was evaluated using time-dependent AUC. The optimal model’s risk score underwent univariate Cox regression (coxph function, survival package) to calculate hazard ratios (HR) with 95% CIs across validation datasets. We performed fixed-effects meta-analysis of log-hazard ratios (logHRs) using inverse variance weighting. Kaplan-Meier survival curves (survival package) compared high/low-risk groups (optimal cutoff determined by survminer; high:low ratio ≥0.3), with statistical significance assessed *via* log-rank tests (survfit function).

### 2.8 Drug sensitivity analysis

Tumor stemness indices—including mRNA expression-based (RNAss) and epigenetically regulated RNA expression-based (EREG.EXPss) indices—were sourced from prior studies (Malta et al., 2018). Spearman correlations between gene expression and drug sensitivity (quantified by dose-response curve AUC) were computed using Cancer Therapeutics Response Portal (CTRP) and Profiling Relative Inhibition Simultaneously in Mixtures (PRISM) databases. Negative correlations indicated increased drug sensitivity with higher expression, while positive correlations denoted greater drug resistance. Per Yang et al. (Malta et al., 2018), the eXtreme Sum (XSum) method optimally extracts biologically meaningful results from Connectivity Map (CMap) analysis. To identify compounds counteracting HCCS-mediated tumor promotion, we performed CMap analysis. Using XSum for signature matching, HCCS-related gene signatures were compared against CMap references to generate similarity scores for 1,288 compounds, following established protocols (Malta et al., 2018; Yang et al., 2022). Lower-scoring compounds represent candidates for inhibiting HCCS-driven oncogenesis.

### 2.9 Cell culture and maintenance

The human breast cancer cell line MDA-MB-231 was acquired from Pricella Biotechnology (Wuhan, China). Cells were maintained in Dulbecco’s Modified Eagle Medium (DMEM; HyClone, USA) containing 10% fetal bovine serum (FBS; Vazyme, China) and 1% penicillin-streptomycin (100 U/mL and 100 mg/mL, respectively). Standard incubation conditions were employed at 37 °C in a humidified atmosphere with 5% CO_2_.

### 2.10 Quantitative real-time PCR (qRT-PCR)

Total RNA was extracted using TRIzol reagent (Invitrogen, USA) following the manufacturer’s protocol. For cDNA synthesis, 1 μg of purified RNA was reverse-transcribed utilizing the HiScript III first Strand cDNA Synthesis Kit (Vazyme, China). Quantitative PCR was subsequently performed using ChamQ Universal SYBR qPCR Master Mix (Vazyme, China). Each 15.4 μL reaction volume comprised 1 μL of cDNA template, 0.6 μL of each primer (10 μM), 7.5 μL of Master Mix, and 6.3 μL of nuclease-free water. The thermal cycling profile included an initial denaturation at 95 °C for 10 min, followed by 40 cycles of 95 °C for 15 s, 62 °C for 60 s, and 72 °C for 15 s. A final melt curve analysis was conducted starting at 60 °C. GAPDH served as the endogenous control for normalization. Primer sequences were as follows: GAPDH (F: 5′-GTC​TCC​TCT​GAC​TTC​AAC​AGC​G-3′, R: 5′-ACC​ACC​CTG​TTG​CTG​TAG​CCA​A-3′) and HCCS (F: 5′-GTA​CGT​GGA​GTG​TCC​CAT​TAG​G-3′, R: 5′-CTG​CTC​TCG​GAA​TGG​ATG​ACT​C-3′).

### 2.11 Western blotting

Post-siRNA transfection, MDA-MB-231 cells were harvested and rinsed thrice with ice-cold phosphate-buffered saline (PBS). Total protein was extracted using RIPA lysis buffer supplemented with protease inhibitors (Solarbio, China). For immunoblotting, membranes were incubated with primary antibodies against HCCS and GAPDH (Proteintech, China) according to the manufacturer’s protocols. Subsequently, the membranes were treated with a goat anti-rabbit IgG-HRP secondary antibody (Proteintech, China). GAPDH served as the internal loading control. Protein bands were visualized using an enhanced chemiluminescence (ECL) detection kit (4A Biotech, China).

### 2.12 Flow cytometric analysis of cell cycle

To quantify cell cycle progression, MDA-MB-231 cells were harvested and fixed in pre-chilled 70% ethanol at 4 °C overnight. After being rinsed twice with phosphate-buffered saline (PBS), the cells were subjected to DNA staining using a PI/RNase reagent (Cell Cycle Detection Kit; KeyGen Biotech, China) for 30 min. Data acquisition was performed on a Beckman flow cytometer, and the proportion of cells in each phase was determined using CytExpert Software.

### 2.13 Cell proliferation and colony formation analysis

Cell growth dynamics were quantified using the CCK-8 assay (APExBIO, USA) and a clonogenic survival model. Briefly, HCCS-silenced MDA-MB-231 cells were inoculated into 96-well plates (5,000 cells/well). Absorbance was measured at four consecutive time points (0, 24, 48, and 72 h) post-incubation. Parallelly, the clonogenic capacity was assessed by seeding 1 × 10^3^ cells/well into 12-well plates. After a growth period of 1–2 weeks post-transfection, the resulting macro-colonies were immobilized with 4% paraformaldehyde and stained with crystal violet. The proliferation index and colony numbers were subsequently processed and analyzed using ImageJ software to determine the inhibitory effects of HCCS silencing.

### 2.14 Statistical analysis

All analyses were conducted in R (v4.3.3) or *via* public databases. Group comparisons of categorical variables (e.g., tumor vs. normal) employed the Wilcoxon rank-sum test. For >2 groups, the Kruskal–Wallis test assessed significance. Correlation analyses used: 1) Pearson (cor.test, method = 'pearson’) for normally distributed or dimensionally matched data; 2) Spearman (cor.test, method = 'spearman’) for non-normal or dimensionally mismatched data. Chi-square tests (chisq.test) analyzed contingency tables. Diagnostic performance was evaluated *via* ROC curves using the pROC package. To control the false discovery rate (FDR) in large-scale screening (e.g., DEG and GSEA), p-values were adjusted using the Benjamini–Hochberg method, and an adjusted p < 0.05 was considered statistically significant. Data from cellular experiments were analyzed using GraphPad Prism for Windows (version 9.0.0). Statistical significance was set at *p* < 0.05, with *p* < 0.05 indicated by one asterisk (*), *p* < 0.01 by two asterisks (**), *p* < 0.001 by three asterisks (***), and ‘ns’ denoting not significant.

## 3 Results

### 3.1 HCCS dysregulation in multiple tumors

**FIGURE 1:**
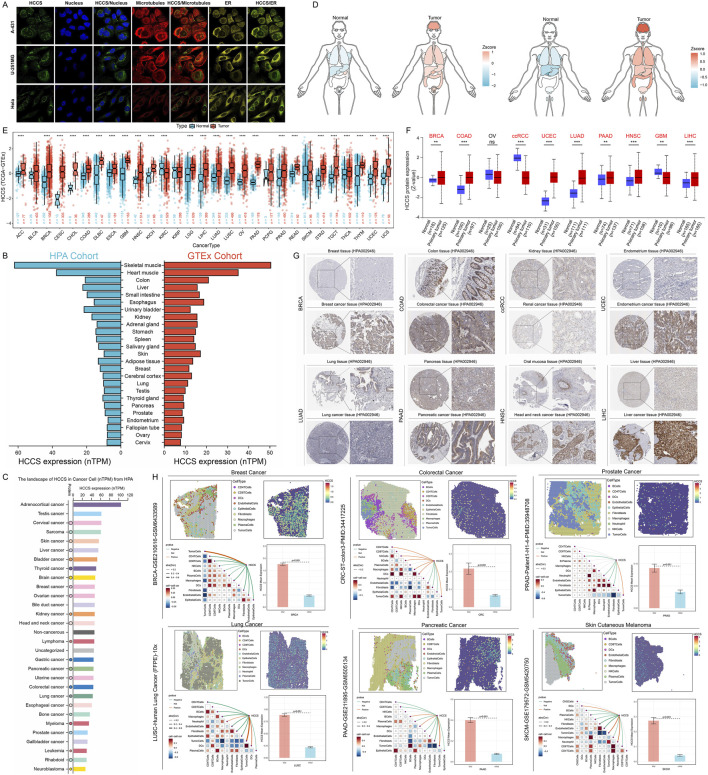
Expression Landscape and Subcellular Localization of HCCS Across Human Tissues and Tumors. **(A)** Immunofluorescence images from the Human Protein Atlas (HPA) database illustrating the subcellular localization of HCCS protein. **(B)** mRNA expression levels of HCCS across various normal human tissues were integrated from HPA and GTEx databases. **(C)** Analysis of HCCS expression profiles across various cancer cell lines based on the HPA database. **(D)** Organogram representing the differential expression of HCCS between normal and tumor tissues. **(E)** Comparison of HCCS mRNA expression between tumor and adjacent normal tissues in the TCGA Pan-Cancer cohort. **(F)** Differential HCCS protein expression analysis across multiple cancer types using CPTAC data. **(G)** Representative immunohistochemistry (IHC) staining from the HPA database validating HCCS protein expression differences in various tissues. **(H)** Spatial transcriptomic analysis revealing HCCS expression patterns within the tumor microenvironment and its enrichment in specific malignant cell clusters.

Subcellular localization analysis confirmed mitochondrial expression of HCCS (Figure 1A; Supplementary Figure S1A). In normal human tissues, HCCS exhibited highest expression in skeletal and cardiac muscle (Figure 1B). Tumor cell line profiling revealed elevated HCCS expression in adrenocortical carcinoma (ACC), testicular germ cell tumors (TGCT), and cervical squamous cell carcinoma (CESC) lines (Figure 1C), while immune cell analysis showed predominant expression in monocytes and dendritic cells (Supplementary Figure S1B). Pan-cancer transcript analysis demonstrated significant HCCS overexpression in tumors *versus* normal tissues (Supplementary Figure S1C). Differential analysis of The Cancer Genome Atlas (TCGA) samples confirmed upregulation in breast invasive carcinoma (BRCA), kidney chromophobe (KICH), lung adenocarcinoma (LUAD), and uterine corpus endometrial carcinoma (UCEC) (Supplementary Figure S1D,E). Integration with GTEx normal samples revealed HCCS overexpression in >80% of tumor types (Figures 1D,E). This result was fully validated on additional GEO datasets (Supplementary Figure S1F). Proteomic analysis showed elevated HCCS protein levels in BRCA, colon adenocarcinoma (COAD), UCEC, LUAD, PAAD, head and neck squamous cell carcinoma (HNSC), and liver hepatocellular carcinoma (LIHC) (Figure 1F), validated by Human Protein Atlas (HPA) immunohistochemistry (Figure 1G; Supplementary Figure S1G). Spatial transcriptomics revealed HCCS localization patterns strongly correlating with malignant cells, concentrated within discrete tumor cell clusters (Figure 1H). Malignant regions (Mal) exhibited significantly higher HCCS expression than non-malignant regions (nMal) (Supplementary Figure S2A). Single-cell profiling confirmed predominant HCCS overexpression in malignant cells across malignancies (Supplementary Figure S2B), implicating HCCS in tumorigenesis and progression.

### 3.2 Genomic and epigenetic regulation of HCCS expression

**FIGURE 2:**
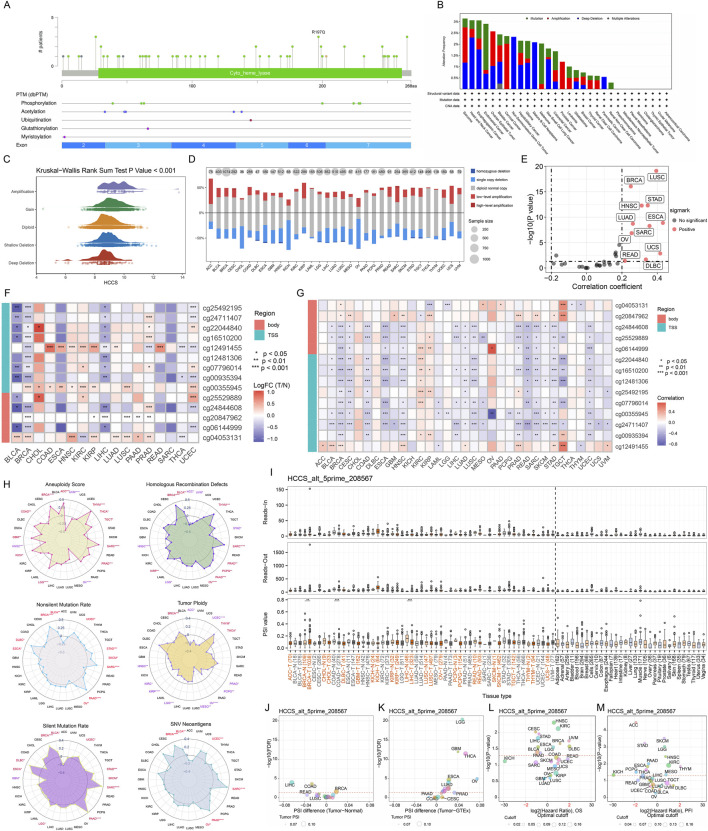
Genomic Alterations, Epigenetic Modifications, and Alternative Splicing of HCCS in Pan-Cancer. **(A)** Lollipop plot depicting the mutation landscape and specific mutation sites of HCCS across the Pan-Cancer cohort. **(B)** Mutation frequency of HCCS across diverse cancer types. **(C,D)** Distribution of HCCS copy number variations (CNV) and their impact on HCCS mRNA expression. **(E)** Correlation between CNV burden and HCCS transcript levels. **(F,G)** Analysis of HCCS promoter methylation levels in tumor vs. normal tissues and its correlation with mRNA expression. **(H)** Correlation between HCCS expression and established Pan-Cancer oncogenic gene set scores. **(I)** Visualization of reads-in, reads-out, and Percent Spliced In (PSI) values for the HCCS_alt_5prime_208567 event across tumors and normal tissues. **(J–M)** Differential analysis of PSI values and the prognostic significance of specific HCCS alternative splicing events.

To elucidate mechanisms underlying HCCS dysregulation, we analyzed mutations, copy number variations (CNVs), and promoter methylation. Figure 2A illustrates the pan-cancer spectrum of HCCS mutations. HCCS amplifications predominated in sarcoma (SARC), ovarian epithelial tumors, prostate adenocarcinoma (PRAD), and CESC, while deep deletions were frequent in HNSC, esophagogastric cancers, non-seminomatous germ cell tumors, and mature B-cell neoplasms. UCEC and melanomas exhibited high single-nucleotide variant rates (Figure 2B). HCCS expression increased progressively from deep deletions to high-copy-number amplifications (Figure 2C). Copy number losses affected HCCS across most tumor types, with gains observed in minority samples (Figure 2D). Significant positive correlations between HCCS mRNA expression and CNV occurred in LUSC, BRCA, stomach adenocarcinoma (STAD), and esophageal carcinoma (ESCA) (Figure 2E; Supplementary Figure S3A). HCCS CNV status showed significant prognostic associations in UCEC, kidney renal papillary cell carcinoma (KIRP), and LUAD (Supplementary Figure S3B,C). Notably, HCCS expression positively correlated with immune response status (Supplementary Figure S3D). Epigenetically, HCCS probes showed significant hypomethylation in tumor *versus* normal tissues of bladder urothelial carcinoma (BLCA), BRCA, LIHC, and UCEC (Figure 2F). Methylation levels at promoter and gene body sites negatively correlated with mRNA expression in BRCA, COAD, ESCA, and LUSC (Figure 2G). Clinically, HCCS hypomethylation predicted poor prognosis in UCEC and ESCA, while hypermethylation associated with adverse outcomes in PAAD (Supplementary Figure S3E,F). Based on established genomic states (Wu et al., 2022), HCCS expression correlated variably with corresponding gene set scores. In BRCA, HCCS showed significant positive correlations with all six genomic state signatures (Figure 2H).

Specific splicing events in tumor cells can drive disease progression, and their identification offers novel biomarkers for patient stratification (Yong et al., 2025). Alternative splicing (AS) events in HCCS demonstrated clinical relevance, with six events identified *via* OncoSplicing (Supplementary Table S1). The HCCS_alt_5prime_208567 event (TCGA SpIAdderSeq) showed significantly increased percent spliced in (PSI) in BRCA but decreased PSI in COAD and LIHC (Figure 2I). Figures 2J–M quantify tumor-normal PSI differences and prognostic relevance, respectively. The HCCS_AD_88468 event (TCGA SpliceSeq) is detailed in Supplementary Figure S3G–J. These findings position HCCS splicing variants as potential drivers of tumor progression.

### 3.3 HCCS modulates tumor cell cycle progression

**FIGURE 3:**
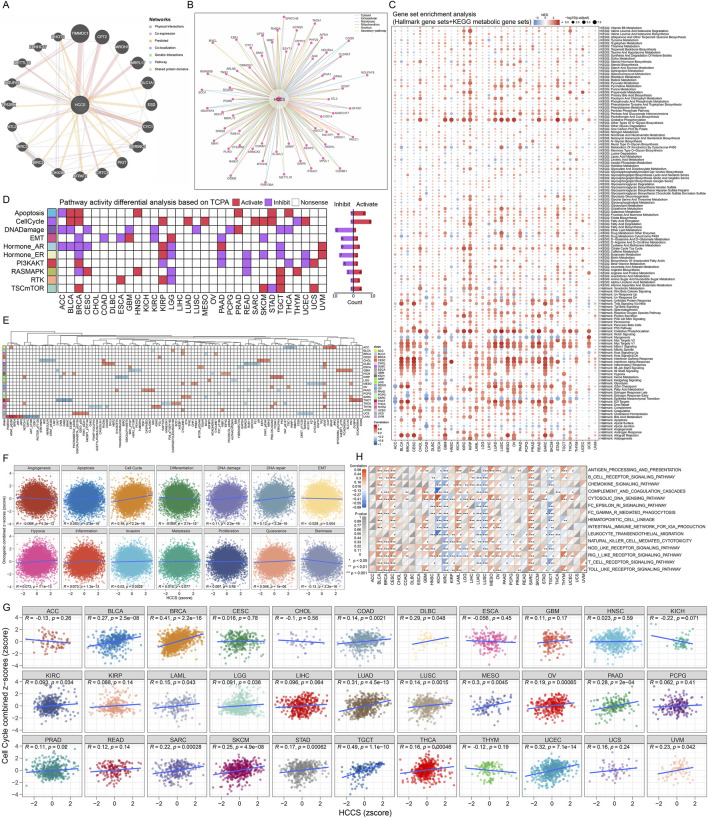
Functional Enrichment and Protein-Protein Interaction (PPI) Networks of HCCS. **(A,B)** Identification of the HCCS-related gene interaction network and potential interacting proteins using GeneMANIA and ComPPI databases. **(C)** Gene Set Enrichment Analysis (GSEA) highlighting pathways associated with HCCS based on Hallmark and KEGG signatures. **(D)** Comparison of pathway activity scores between HCCS-high and HCCS-low expression groups utilizing the TCPA database. **(E)** Heatmap of the top 5 functional proteins significantly correlated with HCCS across cancer types. **(F,G)** Pearson correlation analysis between HCCS expression and malignancy signatures as well as cell cycle scores. **(H)** Correlation heatmap between HCCS expression and inflammation-related gene sets in the Pan-Cancer cohort.

Gene interaction network analysis identified TIMMDC1, CPT2, MROH1, and MRPL17 as primary HCCS interactors (Figure 3A). ComPPI further delineated potential HCCS-binding partners (Figure 3B). GSEA revealed significant activation of: Cell cycle pathways (MTORC1 signaling, MYC targets, G2M checkpoint, E2F targets), Immune pathways (interferon gamma response, inflammatory response), and Metabolic pathways in HCCS-high tumors (Figure 3C; Supplementary Figure S4A–D). Proteomic pathway analysis demonstrated elevated apoptosis and cell cycle activity in HCCS-overexpressing samples (Figure 3D). HCCS mRNA expression positively correlated with cyclin B1 (CCNB1) protein levels in BLCA, BRCA, and SARC (Figure 3E). Pan-cancer functional status analysis confirmed HCCS association with cell cycle progression and DNA damage repair (Figure 3F), with significant correlations in: BRCA (r = 0.41, P < 0.001), LUAD (r = 0.31, P < 0.001), Mesothelioma (MESO) (r = 0.30, P = 0.005), Testicular germ cell tumors (TGCT) (r = 0.49, P < 0.001), Uterine corpus endometrial carcinoma (UCEC) (r = 0.32, P < 0.001) (Figure 3G). Notably, further analysis revealed that HCCS expression was not strongly correlated with the global proliferation signature across most TCGA types (Supplementary Figure S4E), suggesting its effects are distinct from generic cell proliferation programs. Furthermore, HCCS expression positively correlated with immune-inflammatory pathways across multiple malignancies including BLCA, BRCA, CESC, acute myeloid leukemia (LAML), and SARC (Figure 3H).

### 3.4 Pan-cancer immunological signatures of HCCS

**FIGURE 4:**
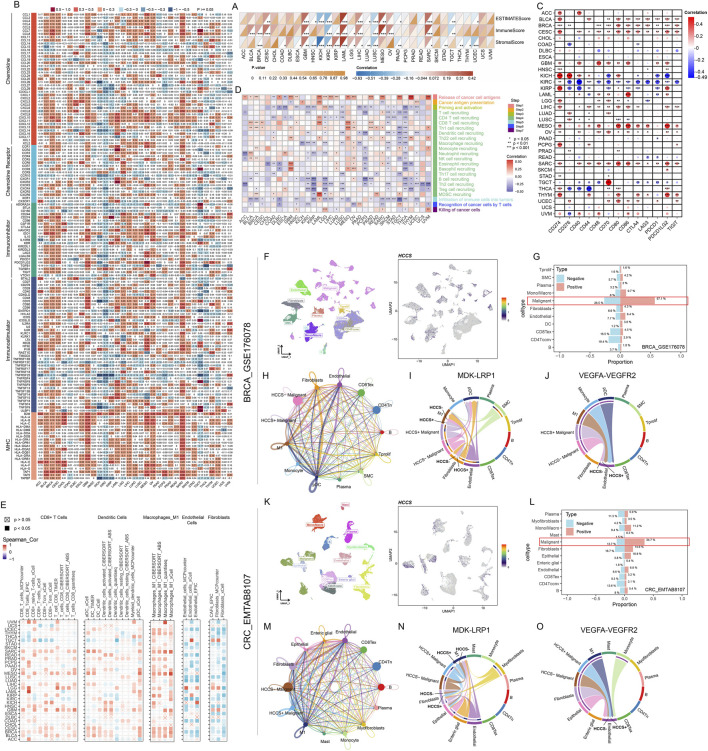
The Role of HCCS in the Tumor Immune Microenvironment and Intercellular Communication. **(A)** Evaluation of the correlation between HCCS expression and immune/stromal scores calculated by the ESTIMATE algorithm. **(B,C)** Correlation analysis of HCCS with immune-regulatory genes and checkpoint molecules. **(D)** Heatmap showing the association between HCCS and the seven steps of the cancer-immunity cycle. **(E)** Multi-algorithm immune infiltration analysis (e.g., TIMER, CIBERSORT). correlating HCCS with CD8^+^ T cells, DCs, macrophages, and fibroblasts. **(F–K)** Single-cell RNA sequencing (scRNA-seq) analysis of BRCA (GSE176078) and CRC (EMTAB8107) cohorts demonstrating HCCS localization in malignant and myeloid subsets. **(G–L)** Cellular composition shifts in HCCS-positive vs. HCCS-negative groups. **(M–O)** Ligand-receptor crosstalk analysis showing HCCS-related malignant cells interacting with the TME *via* MDK-LRP1 and VEGFA-VEGFR2 axes.

HCCS expression positively correlated with ImmuneScore and StromalScore in BRCA, glioblastoma (GBM), LAML, and SARC (Figure 4A). Significant associations with immune-related gene activation were observed in BLCA, BRCA, CESC, GBM, MESO, and SARC (Figure 4B). Immune signature correlation analysis suggested HCCS involvement in immunoregulation (Supplementary Figure S5A). The IFN-γ-dominant (C2) immune subtype predominated in HCCS-high tumors, while inflammatory (C3) subtypes were enriched in HCCS-low groups (Supplementary Figure S5B,C). HCCS expression positively correlated with immune checkpoint genes (CD274, CTLA4) in BRCA, CESC, GBM, MESO, and SARC (Figure 4C). Analysis of the tumor-immune cycle revealed a significant positive correlation between HCCS and the release of cancer cell antigens (step 1) and a significant negative correlation with the infiltration of immune cells into tumors (step 5), a finding that was consistent across multiple tumor types (Figure 4D; Supplementary Figure S5D,E). This finding highlights the potential link between HCCS and immunotherapy. Analysis of immune cell infiltration using multiple algorithms revealed that HCCS reduced the infiltration of endothelial cells and fibroblasts while promoting the infiltration of CD8^+^ T cells, dendritic cells, and macrophages_M1 (Figure 4E). Single-cell analyses of BRCA (GSE176078) and CRC (EMTAB8107) confirmed that HCCS expression was primarily localized to malignant cells, macrophages, endothelial cells, and fibroblasts (Figures 4F,K). Cell proportion analysis revealed that malignant cells accounted for a significant proportion of the HCCS-positive group (Figures 4G,L), suggesting that malignant cells may be the primary contributor to HCCS expression. As shown in Figures 4H,M, the network diagram clearly demonstrates the number and strength of the communication network. Analysis of BRCA ligand-receptor pathways revealed that HCCS + malignant tumor cells exerted significantly stronger regulatory effects on M1 macrophages and fibroblasts *via* the MDK-LRP1 axis and on endothelial cells *via* the VEGFA-VEGFR2 axis than HCCS- cells (Figures 4I,J). Similar results were observed in CRC (Figures 4N,O).

### 3.5 HCCS predicts immunotherapy response

**FIGURE 5:**
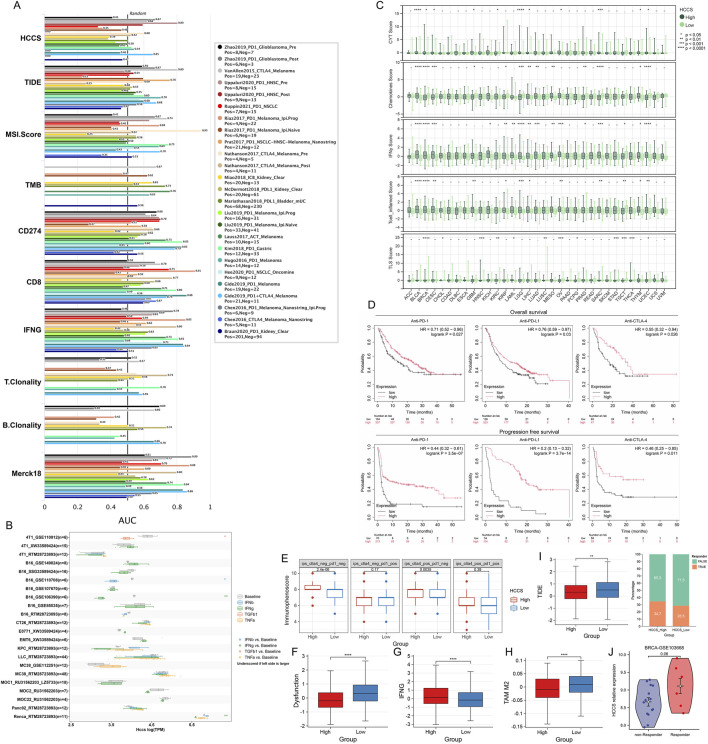
HCCS as a Predictive Biomarker for Immunotherapy Response. **(A)** Performance comparison of HCCS against established biomarkers (e.g., TIDE, MSI) in predicting immunotherapy outcomes. **(B)** Analysis of HCCS expression in therapy responders vs. non-responders using the TISMO database. **(C)** Evaluation of Pan-Cancer immune signatures (CYT, TLS, IFNγ) using the easier R package. **(D)** Kaplan-Meier analysis of HCCS expression in relation to survival in patients receiving immune checkpoint inhibitors (ICI). **(E)** Correlation of HCCS with anti-PD1/anti-CTLA4 therapeutic response in BRCA (TCIA database). **(F–I)** Association between HCCS expression and TIDE-related dysfunction and exclusion scores. **(J)** Validation of HCCS expression differences in the GSE103668 BRCA immunotherapy cohort.

Comparative biomarker analysis positioned HCCS as a potential predictive biomarker (Figure 5A). Using the Tumor Immune Syngeneic MOuse (TISMO) tool, cytokine-treated cell lines showed significantly reduced HCCS expression in therapy responders (Figure 5B). Through EaSIeR—a biomarker platform predicting immune checkpoint inhibitor (ICI) response—patients with high HCCS expression exhibited elevated IFNγ and T-cell-inflamed scores across BLCA, BRCA, and CESC (Figure 5C), suggesting enhanced antigen presentation and T-cell recruitment. Survival analysis confirmed that high-HCCS patients had significantly longer OS and progression-free survival (PFS) following anti-PD-1, anti-PD-L1, or anti-CTLA-4 therapy (Figure 5D). Given strong breast cancer associations, we analyzed BRCA-specific immunotherapy relevance: High-HCCS patients showed significantly higher IPS values in both baseline (PD-1^−^CTLA-4^-^) and anti-CTLA-4 monotherapy (PD-1^−^CTLA-4^+^) groups (Figure 5E). TIDE uses a panel of gene expression markers to assess two distinct mechanisms of tumor immune escape: dysfunction of tumor-infiltrating cytotoxic T lymphocytes (CTLs) and rejection of CTLs by immunosuppressive factors. TIDE algorithm results showed that patients with high HCCS expression in BRCA had lower Dysfunction scores and TAM M2 scores, but higher IFNG scores (Figures 5F–H). Critically, high-HCCS patients had lower TIDE scores, superior immune checkpoint blockade (ICB) efficacy, and increased responder frequency, predicting prolonged post-ICB survival (Figure 5I). In triple-negative breast cancer (GSE103668; cisplatin/bevacizumab cohort), responders showed a non-significant trend toward higher HCCS expression (Figure 5J).

### 3.6 Pan-cancer diagnostic and prognostic value of HCCS

**FIGURE 6:**
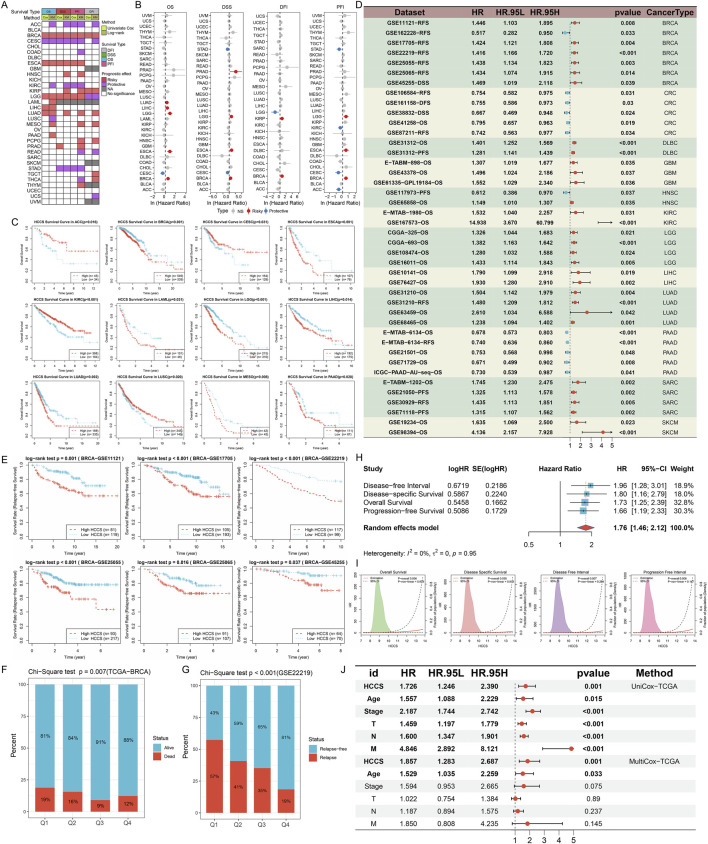
Pan-Cancer Prognostic Value of HCCS and Focused Validation in Breast Cancer. **(A)** Heatmap summarizing the association between HCCS expression and multiple survival endpoints (OS, DSS, PFI, DFI) across cancer types. **(B)** Forest plots of univariate Cox regression analysis for HCCS-related prognosis. **(C–E)** Kaplan-Meier survival curves for OS across pan-cancer and specific BRCA datasets. **(F,G)** Survival status distribution across HCCS expression quartiles (Q1-Q4) in BRCA patients. **(H)** Meta-analysis integrating multiple datasets to confirm the prognostic impact of HCCS in BRCA. **(I)** Restricted cubic splines (RCS) analysis showing the non-linear relationship between HCCS levels and survival risk. **(J)** Multivariate Cox regression identifying HCCS as an independent prognostic factor.

ROC curve analysis demonstrated that HCCS exhibited significant diagnostic performance across a wide range of cancer types, particularly CESC, KICH, Kidney renal clear cell carcinoma (KIRC), LUSC, PAAD, and UCEC. In terms of diagnostic value, HCCS demonstrates significant clinical utility: it exhibits excellent diagnostic performance in cancers such as CESC (AUC: 0.995; 95% CI: 0.984–1.000) and KICH (AUC: 0.963; 95% CI: 0.917–0.998) (Supplementary Figure S6A,B). High AUC values indicate that HCCS can effectively discriminate between tumor and normal tissue, suggesting its potential as a pan-cancer diagnostic marker. Subsequently, we evaluated the prognostic relevance of HCCS as a predictor across 33 tumor types. A heat map illustrates the association of HCCS with different survival outcomes across the pan-cancer spectrum (Figure 6A). Univariate Cox regression analysis demonstrated that high HCCS gene expression was associated with a significantly increased risk of overall survival mortality in patients with BRCA, ESCA, Lower Grade Glioma (LGG), LIHC, and LUAD. Conversely, in CESC, HCCS gene expression was associated with a decreased risk of overall survival mortality. HCCS was also a risk factor for poorer DSS in BRCA, ESCA, LGG, and PRAD, whereas it was a protective factor in CESC and STAD. Regarding the PFI, HCCS was a risk factor in BRCA, ESCA, and LGG, while it had a protective effect in ACC, CESC, KIRC, and STAD. Regarding the DFI, HCCS was a risk factor in BRCA and KIRP, while it had a protective effect in LGG (Figure 6B). Kaplan-Meier curves were also used to assess these four prognostic outcomes (Figure 6C; Supplementary Figure S6C). Overall, these results indicate that higher levels of HCCS expression are generally associated with worse prognosis in patients with BRCA, ESCA, KIRP, LUAD, and MESO. To further validate the clinical prognostic value of HCCS, we collected multiple tumor datasets with clinical data. Univariate Cox survival analysis and Kaplan-Meier survival analysis showed that high HCCS expression was significantly associated with poor prognosis in multiple cancer types, including BRCA, LGG, LIHC, and LUAD (Figures 6D,E; Supplementary Figure S7A–L). Given the consistent correlation between high HCCS expression and poor BRCA prognosis across multiple datasets, we further analyzed BRCA-specific data. Statistical analysis based on the chi-square test revealed that the 25% of BRCA patients with the highest HCCS expression (Q1 group) had significantly more deaths than the other groups (Figures 6F,G). Meta-analysis revealed that high HCCS gene expression was significantly associated with poor survival in BRCA patients (pooled HR = 1.76, 95% CI: [1.46, 2.12], p < 0.05) (Figure 6H). This result suggests that HCCS gene expression levels may serve as potential biomarkers for prognostic assessment in BRCA patients. RCS analysis demonstrated a linear effect of HCCS gene expression on survival risk in BRCA patients (Figure 6I). In multivariate Cox regression analysis incorporating multiple clinical variables, HCCS gene expression demonstrated statistical significance (p = 0.001), confirming its role as an independent prognostic factor (Figure 6J).

### 3.7 HCCS is significantly associated with multiple BRCA clinical features

**FIGURE 7:**
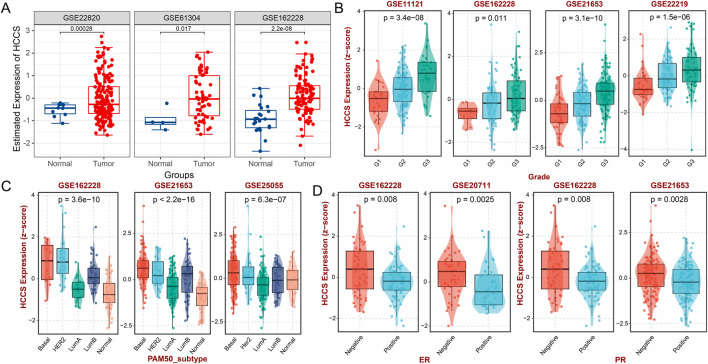
Clinicopathological Significance of HCCS in Breast Cancer. **(A)** Validation of HCCS upregulation in BRCA using independent external cohorts. **(B–D)** Correlation of HCCS expression with clinical Grade, molecular subtypes (e.g., Basal-like), and hormone receptor (ER/PR) status.

Differential analysis across multiple datasets demonstrated significant overexpression of HCCS in BRCA tumor tissues (Figure 7A). Importantly, HCCS expression increased with advancing histologic grade in BRCA patients (Figure 7B), underscoring its potential significance in tumor development and progression. Subtype analysis revealed significantly elevated HCCS expression in the Basal subtype of BRCA (Figure 7C). Furthermore, HCCS was significantly overexpressed in estrogen receptor (ER)-negative and progesterone receptor (PR)-negative BRCA patients (Figure 7D). To further explore associations between HCCS and BRCA clinical features, we analyzed 13 BRCA datasets containing clinical information. Chi-square tests demonstrated significant correlations between HCCS expression levels and multiple clinical stage features within these datasets (Table 1). These results suggest HCCS expression plays a crucial role in tumor progression, providing a foundation for further investigation of its tumorigenic mechanisms.

**TABLE 1:** Systematic correlation analysis between HCCS levels and diverse clinicopathological features across multiple BRCA datasets.

Dataset	Clinical	Type	Total	HCCS_High	HCCS_Low	X-squared	Pvalue
**TCGA**	Age	>50	733 (69.35%)	371 (69.87%)	362 (68.82%)	0.0914	0.7624
​	​	≤50	324 (30.65%)	160 (30.13%)	164 (31.18%)	​	​
​	Stage	I	175 (16.56%)	83 (15.63%)	92 (17.49%)	2.7785	0.4271
​	​	II	598 (56.58%)	306 (57.63%)	292 (55.51%)	​	​
​	​	III	241 (22.8%)	117 (22.03%)	124 (23.57%)	​	​
​	​	IV	20 (1.89%)	13 (2.45%)	7 (1.33%)	​	​
​	​	Unknown	23 (2.18%)	12 (2.26%)	11 (2.09%)	​	​
​	T	T1	273 (25.83%)	129 (24.29%)	144 (27.38%)	13.4962	**0.0037**
​	​	T2	614 (58.09%)	327 (61.58%)	287 (54.56%)	​	​
​	​	T3	129 (12.2%)	49 (9.23%)	80 (15.21%)	​	​
​	​	T4	38 (3.6%)	24 (4.52%)	14 (2.66%)	​	​
​	​	Unknown	3 (0.28%)	2 (0.38%)	1 (0.19%)	​	​
​	N	N0	492 (46.55%)	240 (45.2%)	252 (47.91%)	2.4418	0.4859
​	​	N1	352 (33.3%)	181 (34.09%)	171 (32.51%)	​	​
​	​	N2	118 (11.16%)	65 (12.24%)	53 (10.08%)	​	​
​	​	N3	75 (7.1%)	34 (6.4%)	41 (7.79%)	​	​
​	​	Unknown	20 (1.89%)	11 (2.07%)	9 (1.71%)	​	​
​	M	M0	877 (82.97%)	461 (86.82%)	416 (79.09%)	1.5207	0.2175
​	​	M1	22 (2.08%)	15 (2.82%)	7 (1.33%)	​	​
​	​	Unknown	158 (14.95%)	55 (10.36%)	103 (19.58%)	​	​
**GSE162228**	Age	>50	64 (58.72%)	41 (65.08%)	23 (50%)	1.9107	0.1669
​	​	≤50	45 (41.28%)	22 (34.92%)	23 (50%)	​	​
​	Grade	G1	6 (5.5%)	2 (3.17%)	4 (8.7%)	5.2207	0.0735
​	​	G2	52 (47.71%)	26 (41.27%)	26 (56.52%)	​	​
​	​	G3	51 (46.79%)	35 (55.56%)	16 (34.78%)	​	​
​	Stage	I	24 (22.02%)	14 (22.22%)	10 (21.74%)	8.7254	**0.0332**
​	​	II	46 (42.2%)	32 (50.79%)	14 (30.43%)	​	​
​	​	III	29 (26.61%)	13 (20.63%)	16 (34.78%)	​	​
​	​	IV	3 (2.75%)	0 (0%)	3 (6.52%)	​	​
​	​	Unknown	7 (6.42%)	4 (6.35%)	3 (6.52%)	​	​
**GSE20685**	Age	>50	118 (36.09%)	63 (38.89%)	55 (33.33%)	0.8663	0.3520
​	​	≤50	209 (63.91%)	99 (61.11%)	110 (66.67%)	​	​
​	T	T1	101 (30.89%)	40 (24.69%)	61 (36.97%)	8.4192	**0.0381**
​	​	T2	188 (57.49%)	102 (62.96%)	86 (52.12%)	​	​
​	​	T3	26 (7.95%)	16 (9.88%)	10 (6.06%)	​	​
​	​	T4	12 (3.67%)	4 (2.47%)	8 (4.85%)	​	​
​	N	N0	137 (41.9%)	61 (37.65%)	76 (46.06%)	3.589	0.3094
​	​	N1	87 (26.61%)	48 (29.63%)	39 (23.64%)	​	​
​	​	N2	63 (19.27%)	30 (18.52%)	33 (20%)	​	​
​	​	N3	40 (12.23%)	23 (14.2%)	17 (10.3%)	​	​
​	M	M0	319 (97.55%)	160 (98.77%)	159 (96.36%)	1.0976	0.2948
​	​	M1	8 (2.45%)	2 (1.23%)	6 (3.64%)	​	​
**GSE20711**	Age	>50	61 (69.32%)	33 (73.33%)	28 (65.12%)	0.3652	0.5456
​	​	≤50	27 (30.68%)	12 (26.67%)	15 (34.88%)	​	​
​	Grade	G1	13 (14.77%)	1 (2.22%)	12 (27.91%)	11.5253	**0.0031**
​	​	G2	5 (5.68%)	3 (6.67%)	2 (4.65%)	​	​
​	​	G3	70 (79.55%)	41 (91.11%)	29 (67.44%)	​	​
**GSE21653**	Age	>50	163 (61.28%)	84 (63.16%)	79 (59.4%)	0.3395	0.5601
​	​	≤50	102 (38.35%)	48 (36.09%)	54 (40.6%)	​	​
​	​	Unknown	1 (0.38%)	1 (0.75%)	0 (0%)	​	​
​	Grade	G1	45 (16.92%)	10 (7.52%)	35 (26.32%)	31.1784	**0.0001**
​	​	G2	89 (33.46%)	37 (27.82%)	52 (39.1%)	​	​
​	​	G3	125 (46.99%)	84 (63.16%)	41 (30.83%)	​	​
​	​	Unknown	7 (2.63%)	2 (1.5%)	5 (3.76%)	​	​
​	T	T1	59 (22.18%)	22 (16.54%)	37 (27.82%)	6.8855	**0.0320**
​	​	T2	126 (47.37%)	66 (49.62%)	60 (45.11%)	​	​
​	​	T3	68 (25.56%)	41 (30.83%)	27 (20.3%)	​	​
​	​	Unknown	13 (4.89%)	4 (3.01%)	9 (6.77%)	​	​
​	N	N0	120 (45.11%)	62 (46.62%)	58 (43.61%)	0.1393	0.7090
​	​	N1	140 (52.63%)	68 (51.13%)	72 (54.14%)	​	​
​	​	Unknown	6 (2.26%)	3 (2.26%)	3 (2.26%)	​	​
**GSE22219**	Age	>50	141 (65.28%)	68 (62.96%)	73 (67.59%)	0.3268	0.5675
​	​	≤50	75 (34.72%)	40 (37.04%)	35 (32.41%)	​	​
​	Grade	G1	41 (18.98%)	10 (9.26%)	31 (28.7%)	21.1981	**0.0001**
​	​	G2	87 (40.28%)	40 (37.04%)	47 (43.52%)	​	​
​	​	G3	63 (29.17%)	44 (40.74%)	19 (17.59%)	​	​
​	​	Unknown	25 (11.57%)	14 (12.96%)	11 (10.19%)	​	​
**GSE42568**	Age	>50	77 (74.04%)	36 (69.23%)	41 (78.85%)	0.8004	0.3710
​	​	≤50	27 (25.96%)	16 (30.77%)	11 (21.15%)	​	​
​	Grade	G1	11 (10.58%)	4 (7.69%)	7 (13.46%)	11.171	**0.0038**
​	​	G2	40 (38.46%)	13 (25%)	27 (51.92%)	​	​
​	​	G3	53 (50.96%)	35 (67.31%)	18 (34.62%)	​	​
**GSE7390**	Age	>50	56 (28.28%)	27 (27.27%)	29 (29.29%)	0.0249	0.8746
​	​	≤50	142 (71.72%)	72 (72.73%)	70 (70.71%)	​	​
​	Grade	G1	30 (15.15%)	12 (12.12%)	18 (18.18%)	24.7243	0.0001
​	​	G2	83 (41.92%)	28 (28.28%)	55 (55.56%)	​	​
​	​	G3	83 (41.92%)	59 (59.6%)	24 (24.24%)	​	​
​	​	Unknown	2 (1.01%)	0 (0%)	2 (2.02%)	​	​
**GSE45255**	Age	>50	81 (58.27%)	38 (55.07%)	43 (61.43%)	0.3455	0.5567
​	​	≤50	58 (41.73%)	31 (44.93%)	27 (38.57%)	​	​
​	Grade	G1	17 (12.23%)	3 (4.35%)	14 (20%)	8.299	**0.0158**
​	​	G2	52 (37.41%)	26 (37.68%)	26 (37.14%)	​	​
​	​	G3	67 (48.2%)	38 (55.07%)	29 (41.43%)	​	​
​	​	Unknown	3 (2.16%)	2 (2.9%)	1 (1.43%)	​	​
**GSE61304**	Age	>50	38 (65.52%)	19 (65.52%)	19 (65.52%)	0	1.0000
​	​	≤50	20 (34.48%)	10 (34.48%)	10 (34.48%)	​	​
​	Grade	G1	5 (8.62%)	0 (0%)	5 (17.24%)	6.5743	**0.0374**
​	​	G2	16 (27.59%)	7 (24.14%)	9 (31.03%)	​	​
​	​	G3	37 (63.79%)	22 (75.86%)	15 (51.72%)	​	​
​	Stage	I	5 (8.62%)	2 (6.9%)	3 (10.34%)	1.5399	0.6731
​	​	II	34 (58.62%)	18 (62.07%)	16 (55.17%)	​	​
​	​	III	18 (31.03%)	8 (27.59%)	10 (34.48%)	​	​
​	​	IV	1 (1.72%)	1 (3.45%)	0 (0%)	​	​
​	T	T1	13 (22.41%)	3 (10.34%)	10 (34.48%)	7.0681	0.0698
​	​	T2	35 (60.34%)	22 (75.86%)	13 (44.83%)	​	​
​	​	T3	8 (13.79%)	4 (13.79%)	4 (13.79%)	​	​
​	​	T4	1 (1.72%)	0 (0%)	1 (3.45%)	​	​
​	​	Unknown	1 (1.72%)	0 (0%)	1 (3.45%)	​	​
​	N	N0	13 (22.41%)	7 (24.14%)	6 (20.69%)	3.7032	0.2953
​	​	N1	22 (37.93%)	12 (41.38%)	10 (34.48%)	​	​
​	​	N2	9 (15.52%)	2 (6.9%)	7 (24.14%)	​	​
​	​	N3	6 (10.34%)	4 (13.79%)	2 (6.9%)	​	​
​	​	Unknown	8 (13.79%)	4 (13.79%)	4 (13.79%)	​	​
**GSE25065**	Age	>50	89 (44.95%)	43 (43.43%)	46 (46.46%)	0.0816	0.7751
​	​	≤50	109 (55.05%)	56 (56.57%)	53 (53.54%)	​	​
​	Grade	G1	13 (6.57%)	2 (2.02%)	11 (11.11%)	8.1677	**0.0168**
​	​	G2	63 (31.82%)	30 (30.3%)	33 (33.33%)	​	​
​	​	G3	108 (54.55%)	61 (61.62%)	47 (47.47%)	​	​
​	​	Unknown	14 (7.07%)	6 (6.06%)	8 (8.08%)	​	​
​	Stage	I	2 (1.01%)	1 (1.01%)	1 (1.01%)	1.0085	0.6040
​	​	II	107 (54.04%)	50 (50.51%)	57 (57.58%)	​	​
​	​	III	89 (44.95%)	48 (48.48%)	41 (41.41%)	​	​
​	T	T0	1 (0.51%)	0 (0%)	1 (1.01%)	5.1692	0.2704
​	​	T1	10 (5.05%)	3 (3.03%)	7 (7.07%)	​	​
​	​	T2	90 (45.45%)	41 (41.41%)	49 (49.49%)	​	​
​	​	T3	71 (35.86%)	41 (41.41%)	30 (30.3%)	​	​
​	​	T4	26 (13.13%)	14 (14.14%)	12 (12.12%)	​	​
​	N	N0	70 (35.35%)	39 (39.39%)	31 (31.31%)	3.5048	0.3201
​	​	N1	91 (45.96%)	39 (39.39%)	52 (52.53%)	​	​
​	​	N2	27 (13.64%)	15 (15.15%)	12 (12.12%)	​	​
​	​	N3	10 (5.05%)	6 (6.06%)	4 (4.04%)	​	​
**GSE25055**	Age	>50	142 (45.81%)	65 (41.94%)	77 (49.68%)	1.5724	0.2099
​	​	≤50	168 (54.19%)	90 (58.06%)	78 (50.32%)	​	​
​	Grade	G1	19 (6.13%)	5 (3.23%)	14 (9.03%)	24.77	**0.0001**
​	​	G2	117 (37.74%)	42 (27.1%)	75 (48.39%)	​	​
​	​	G3	151 (48.71%)	96 (61.94%)	55 (35.48%)	​	​
​	​	G4	15 (4.84%)	8 (5.16%)	7 (4.52%)	​	​
​	​	Unknown	8 (2.58%)	4 (2.58%)	4 (2.58%)	​	​
​	Stage	I	6 (1.94%)	4 (2.58%)	2 (1.29%)	4.0548	0.1317
​	​	II	165 (53.23%)	73 (47.1%)	92 (59.35%)	​	​
​	​	III	135 (43.55%)	74 (47.74%)	61 (39.35%)	​	​
​	​	Unknown	4 (1.29%)	4 (2.58%)	0 (0%)	​	​
​	T	T0	2 (0.65%)	2 (1.29%)	0 (0%)	7.4005	0.1162
​	​	T1	20 (6.45%)	11 (7.1%)	9 (5.81%)	​	​
​	​	T2	165 (53.23%)	74 (47.74%)	91 (58.71%)	​	​
​	​	T3	74 (23.87%)	37 (23.87%)	37 (23.87%)	​	​
​	​	T4	49 (15.81%)	31 (20%)	18 (11.61%)	​	​
​	N	N0	87 (28.06%)	37 (23.87%)	50 (32.26%)	3.7122	0.2943
​	​	N1	153 (49.35%)	79 (50.97%)	74 (47.74%)	​	​
​	​	N2	39 (12.58%)	20 (12.9%)	19 (12.26%)	​	​
​	​	N3	31 (10%)	19 (12.26%)	12 (7.74%)	​	​
**GSE11121**	Grade	G1	29 (14.5%)	6 (6%)	23 (23%)	16.4235	**0.0003**
​	​	G2	136 (68%)	69 (69%)	67 (67%)	​	​
​	​	G3	35 (17.5%)	25 (25%)	10 (10%)	​	​

Bold values indicate statistical significance (P < 0.05).

### 3.8 Knockdown of HCCS in BRCA significantly inhibits cell cycle and cell proliferation

**FIGURE 8:**
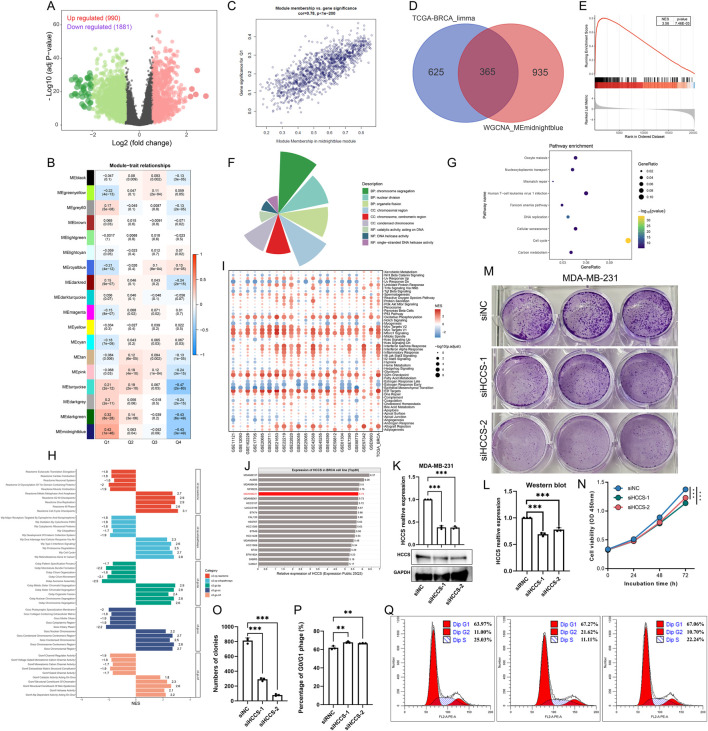
Integrated Bioinformatic Analysis and Experimental Validation of HCCS in BRCA Cell Lines. **(A)** Volcano plot identifying differentially expressed genes (DEGs) between HCCS-high and -low groups. **(B–D)** Weighted Gene Co-expression Network Analysis (WGCNA) identifies the “midnightblue' module and hub genes associated with HCCS. **(E–G)** GO and KEGG enrichment analysis of HCCS-related hub genes. **(H,I)** GSEA confirms the association between HCCS and cell cycle progression. **(J)** Top 20 HCCS expression cell lines in BRCA cell lines. **(K,L)** Efficiency of HCCS knockdown confirmed by qRT-PCR and Western blot in MDA-MB-231 cells. **(M–O)** CCK-8 and colony formation assays demonstrating impaired proliferation following HCCS silencing. **(P,Q)** Flow cytometry analysis showing G0/G1 phase arrest induced by HCCS depletion.

Differential analysis identified 990 genes significantly upregulated in the HCCS high-expression group and 1,881 genes significantly downregulated in the HCCS low-expression group (Figure 8A). WGCNA revealed a significant positive correlation between the MEmidnightblue module gene set and HCCS expression (Figures 8B,C). Integrating these results yielded a set of 365 genes significantly associated with HCCS (Figure 8D). GSEA demonstrated significant enrichment of this 365-gene set in BRCA tumor samples (Figure 8E). Gene Ontology (GO) and KEGG enrichment analyses indicated potential roles for these HCCS-associated genes in cell cycle regulation and DNA repair, providing insights for further investigation (Figures 8F,G). Furthermore, GSEA analysis based on multiple gene sets and multiple BRCA datasets also confirmed a significant correlation between HCCS and the cell cycle of BRCA (Figures 8H,I). To experimentally validate the association between HCCS and the cell cycle in BRCA, we employed the BRCA cell line MDA-MB-231 for functional assays (Figure 8J). Cells were transfected with two distinct siRNAs, followed by qRT-PCR and Western blotting to assess the effects on HCCS expression. Our findings indicated that both mRNA and protein levels of HCCS were reduced in the transfected groups relative to the control groups (Figures 8K,L). Cellular function experiments showed that HCCS silencing significantly inhibited BRCA cell proliferation (Figures 8M–O). Furthermore, HCCS knockdown led to a significant accumulation of G0/G1 phase cells, suggesting that BRCA cell cycle progression and proliferation were suppressed (Figures 8P,Q).

### 3.9 Construction of a consensus prognostic model for BRCA based on HCCS-Related genes

**FIGURE 9:**
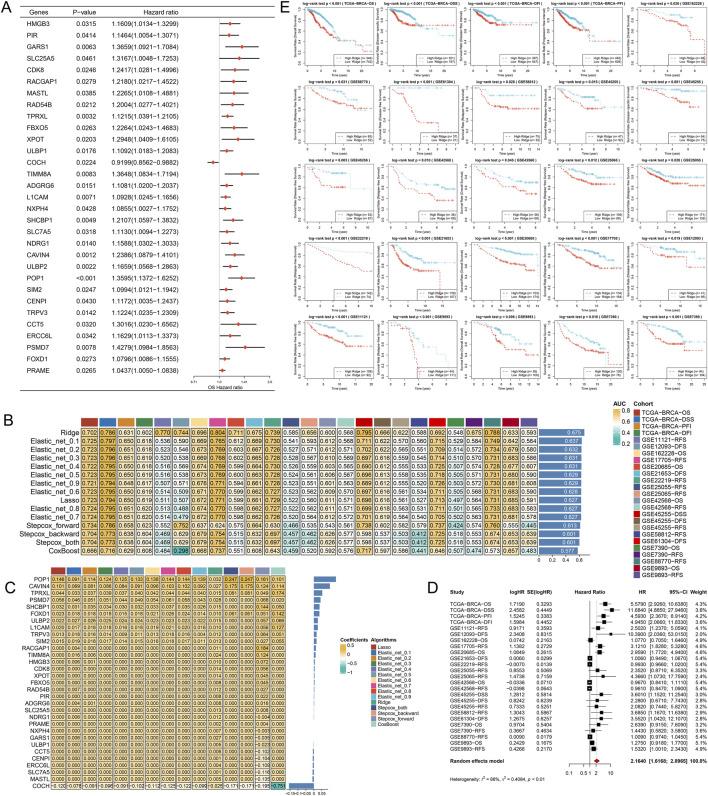
Development and Robustness Evaluation of an HCCS-Based Machine Learning Prognostic Model. **(A)** Selection of 31 candidate prognostic genes *via* univariate Cox analysis. **(B)** Benchmarking of various machine learning algorithms using 1-, 3-, and 5-year AUC values. **(C)** Heatmap of feature coefficients across different modeling algorithms. **(D,E)** Meta-analysis and Kaplan-Meier curves validating the robust predictive performance of the finalized prognostic signature across multiple BRCA cohorts.

Cox survival analysis identified 31 genes significantly associated with OS in BRCA (Figure 9A). We employed multiple algorithms to construct prognostic models and evaluated them comprehensively using the average AUC at 1, 3, and 5 years. The Ridge model demonstrated superior performance with the highest average AUC across all time points and was selected as optimal (Figure 9B). A heatmap displays the regression coefficients of input genes across the different models (Figure 9C). Univariate Cox regression and meta-analysis of the Ridge model risk score confirmed its consistent identification as a risk factor across multiple datasets and survival analyses, indicating high predictive accuracy and generalizability (Figure 9D). Finally, across multiple BRCA cohorts, the high-risk group exhibited significantly worse prognosis compared to the low-risk group (Figure 9E). This validates the reliability and clinical utility of the Ridge model for prognostic assessment and provides important references for future research and application.

### 3.10 HCCS-based prediction of therapeutic drugs and drug sensitivity

**FIGURE 10:**
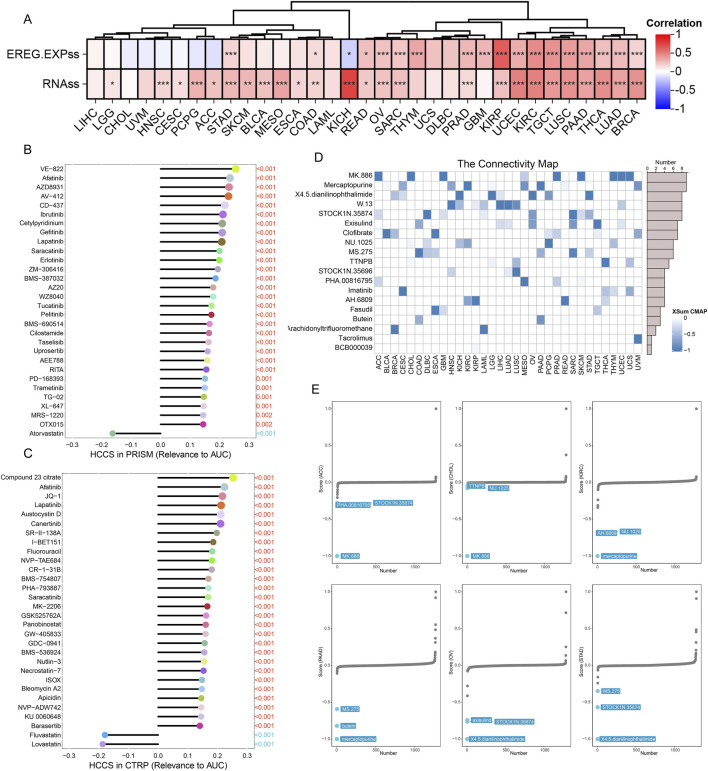
Pharmacogenomic Analysis and Identification of Potential Therapeutic Compounds. **(A)** Correlation between HCCS expression and tumor susceptibility scores. **(B,C)** Drug sensitivity analysis correlating HCCS levels with AUC values in PRISM and CTRP databases. **(D)** Schematic of the XSum algorithm used to identify small molecules from the CMap database that reverse HCCS-associated signatures. **(E)** Top candidate drugs identified (MK.886, mercaptopurine, and X4.5 diphenylamine phthalimide) for targeting the HCCS-driven phenotype.

Stemness scores are considered crucial indicators of drug resistance development and sustained tumor cell proliferation during cancer treatment (Shang et al., 2025). Correlation analysis revealed significant positive associations between HCCS expression and stemness scores in multiple tumor types, including BRCA, LUAD, thyroid carcinoma (THCA), and PAAD (Figure 10A). Analyses using the CTRP and PRISM databases indicated that elevated HCCS expression correlates with resistance to multiple therapeutic agents (Figures 10B,C). Notably, EGFR inhibitors (e.g., lapatinib, afatinib) showed strong positive correlations with HCCS expression in both databases, suggesting HCCS overexpression may confer resistance to EGFR-targeted therapies. Using the CMAP database, we applied the XSum algorithm to predict small molecules potentially counteracting the detrimental effects of dysregulated HCCS expression across pan-cancer datasets (Figure 10D). Results revealed that MK.886, Mercaptopurine, and X4.5.dianilinophthalimide were the top three drugs (Figure 10E).

## 4 Discussion

Mitochondria function as central hubs regulating cellular energy metabolism and apoptosis (Chen et al., 2024). Their dysfunction critically promotes tumorigenesis and cancer progression by disrupting oxidative phosphorylation, exacerbating reactive oxygen species accumulation, and dysregulating apoptotic signaling (San Francisco et al., 2013; Bellah et al., 2025; Mendez et al., 2017). Within this context, cytochrome c (cyt c)—a bifunctional molecule essential for mitochondrial electron transport and apoptosis initiation—undergoes maturation *via* heme attachment catalyzed by holocytochrome c synthase (HCCS) (Babb et al., 2014; Babbitt et al., 2014; Babbitt et al., 2016). While HCCS functional defects are known to cause severe developmental disorders, such as microphthalmia with linear skin defects (MLS) syndrome (Vergult et al., 2013; Wimplinger et al., 2007; Wimplinger et al., 2006; Prakash et al., 2002), and Plasmodium models demonstrate that HCCS deficiency triggers mitochondrial electron transport chain collapse (García-Guerrero et al., 2025), prior research has predominantly focused on HCCS enzymatic mechanisms (e.g., heme-binding domain functions) or single-gene disease associations (Vergult et al., 2013; Grunow et al., 2023; Yeasmin et al., 2024). Consequently, a systematic exploration of HCCS across the pan-cancer spectrum, particularly its association with the tumor immune microenvironment and therapeutic response, has remained lacking. This significant knowledge gap has substantially hindered the clinical translation of HCCS as a therapeutic target.

This study overcomes the limitations of single-cancer-type analyses by systematically mapping the pan-cancer functional landscape of HCCS through the integration of multi-omics data from 33 cancer types and spatial transcriptomic analysis. Unlike previous research confined to *in vitro* enzymatic activity (Babb et al., 2014; Babbitt et al., 2014) or sporadic mutation reports (Wimplinger et al., 2007; Prakash et al., 2002), our breakthrough discovery is the widespread pan-cancer dysregulation of HCCS expression, with over 80% of cancer types exhibiting significant upregulation at both mRNA and protein levels. This dysregulation is driven synergistically by genomic copy number amplification (particularly in SARC and ovarian cancer) and promoter hypomethylation (in BLCA and BRCA). Crucially, spatial transcriptomic analysis revealed, for the first time, specific enrichment of HCCS within malignant tumor regions. Furthermore, HCCS mediates interactions between tumor cells and M2 macrophages and endothelial cells *via* the MDK-LRP1 and VEGFA-VEGFR2 signaling axes. This finding challenges the traditional understanding that HCCS is exclusively localized to the mitochondrial matrix (San Francisco et al., 2013; Babbitt et al., 2016). Beyond its intracellular role in mitochondrial cytochrome c maturation, our data suggest that HCCS orchestrates a ‘mitochondrial-to-secretory’ axis to remodel the tumor microenvironment. Through CellChat inference, we identified HCCS-associated signaling *via* MDK-LRP1 and VEGFA-VEGFR2 axes. This extracellular transition is likely driven by HCCS-mediated metabolic reprogramming; specifically, the activation of MYC and mTORC1 pathways (as evidenced by our GSEA results) is known to enhance the synthesis and secretion of pro-tumorigenic factors like Midkine and VEGFA. Thus, HCCS functions as a metabolic hub that bridges intracellular energetic fitness with extracellular signaling.

At the functional level, this study reveals a dual mechanism by which HCCS drives tumor progression. First, GSEA demonstrated that high HCCS expression activates cell cycle pathways (e.g., G2/M checkpoint, E2F targets) and exhibits significant positive correlation with cyclin B1 (CCNB1) expression, indicating HCCS supports tumor proliferation through cell cycle dysregulation. This finding aligns with reports that HCCS promotes malignant phenotypes in lung cancer models (Luo et al., 2025). Second, HCCS facilitates immune escape by shaping an immunosuppressive microenvironment: elevated HCCS expression increases M2 macrophage infiltration, upregulates immune checkpoint molecules (including CD274 and CTLA4), and activates VEGFA-VEGFR2-mediated angiogenesis. Notably, while earlier studies established that HCCS loss induces apoptotic imbalance (Bellah et al., 2025), they did not investigate its relevance to immune checkpoint blockade therapy.

Clinically, this study establishes HCCS as an innovative predictive biomarker for treatment response. High HCCS expression correlated with enhanced T cell-inflamed gene expression profile (Tcell_inflamed) scores, heightened interferon gamma (IFNγ) signaling, and increased sensitivity to anti-PD-1/CTLA-4 therapy—a finding validated in multiple breast cancer models. Furthermore, drug sensitivity analysis revealed that elevated HCCS expression confers resistance to epidermal growth factor receptor (EGFR) inhibitors (e.g., lapatinib). Critically, the small molecule inhibitor MK-886, identified *via* the CMap database screening, effectively reversed HCCS-mediated tumor-promoting effects, offering a novel strategy to overcome targeted therapy resistance. This advances beyond prior studies focused solely on inhibiting HCCS enzymatic activity (Grunow et al., 2023; Yeasmin et al., 2024). For diagnosis and prognosis, HCCS demonstrated significant clinical utility: it exhibited excellent diagnostic performance in cervical and pancreatic cancers among others. Multicenter cohort analysis confirmed that high HCCS expression significantly increases mortality risk in breast cancer patients (HR = 1.76, 95% CI: 1.46–2.12). Additionally, a machine learning-constructed Ridge regression prognostic model incorporating HCCS-related genes substantially improved breast cancer survival prediction accuracy. In contrast, while earlier studies linked HCCS mutations to microphthalmia (Vergult et al., 2013; Wimplinger et al., 2007), its prognostic potential in cancer remained unexplored.

Pharmacogenomic analysis identified MK-886 as a top candidate for reversing HCCS-associated signatures. Notably, MK-886 exhibits FLAP-independent mitochondrial toxicity—including mitochondrial membrane potential loss, ROS induction, and Bcl-2 inhibition—leading to apoptosis (Anderson et al., 2002). Since HCCS is vital for mitochondrial respiration, these disruptive effects provide a robust mechanistic link between MK-886 and HCCS-mediated immunometabolism, reinforcing the validity of our CMap-driven findings.

Despite the robust evidence presented in this study, several limitations warrant discussion. First, while the cell cycle effects were validated *in vitro*, the immune microenvironment findings are currently correlative and require future *in vivo* validation to establish true causality. Specifically, the causality within the MDK-LRP1 axis necessitates further verification in immunocompetent animal models. Second, due to the absence of a large-scale prospective immunotherapy cohort, the optimal diagnostic cut-off values for HCCS as a companion biomarker remain to be established through rigorous clinical trials. Future investigations will prioritize elucidating the specific metabolic signaling pathways modulated by HCCS within the secretome and evaluating the *in vivo* synergistic efficacy of HCCS inhibitors combined with immunotherapy. In summary, this study not only expands the current understanding of mitochondrial protein functionality but also provides novel targets and a theoretical rationale for the precision diagnosis and treatment of malignant tumors.

## 5 Conclusion

Through multidimensional pan-cancer analysis, this study redefines HCCS as a mitochondrial-immunometabolism hub. Its dysregulated expression—driven by genomic and epigenetic alterations—promotes malignancy *via* cell cycle activation and immunosuppressive microenvironment remodeling. We further demonstrate that HCCS-high tumors exhibit sensitivity to immune checkpoint inhibitors, while small-molecule targeting of the HCCS pathway (e.g., MK-886) may reverse therapeutic resistance. These findings forge crucial links between mitochondrial enzymology and clinical oncology, providing a conceptual framework for mitochondrial-based combination therapies.

## Data Availability

The original contributions presented in the study are publicly available. This data can be found at the NCBI Gene Expression Omnibus (GEO) (https://www.ncbi.nlm.nih.gov/geo/) with the accession numbers: GSE210616, GSE211895, GSE179572, GSE176078, GSE103668, GSE162228, GSE20685, GSE20711, GSE21653, GSE22219, GSE42568, GSE7390, GSE45255, GSE61304, GSE25065, GSE25055, and GSE11121. The CRC single-cell dataset can be found at ArrayExpress (https://www.ebi.ac.uk/arrayexpress/) under accession number E-MTAB-8107. The computer code used in this study has been released at: https://github.com/Time-lee/HCCS. Further inquiries can be directed to the corresponding author.
