# Supplementary material for: Multi-omics landscape and functional validation of HCCS in breast cancer: from pan-cancer immunometabolic characterization to regulating tumor proliferation
Source: Front Genet. 2026 May 19;17:1777429. doi: 10.3389/fgene.2026.1777429 (PMC13225780; doi:10.3389/fgene.2026.1777429)
Supplement: Supplementary file 1 [file DataSheet1.docx]

Supplementary Material

# Supplementary Table

Table S1. Six variable shearing events were identified using OncoSplicing.

| Gene_Symbol | Splice_Type | Splice_Event | Project |
| --- | --- | --- | --- |
| HCCS | AD | HCCS_AD_88468 | SpliceSeq |
| HCCS | AD | HCCS_AD_88469 | SpliceSeq |
| HCCS | AD | HCCS_AD_88470 | SpliceSeq |
| HCCS | A5 | alt_5prime_208564 | SpIAdder |
| HCCS | A5 | alt_5prime_208565 | SpIAdder |
| HCCS | A5 | alt_5prime_208567 | SpIAdder |

# Supplementary Figures


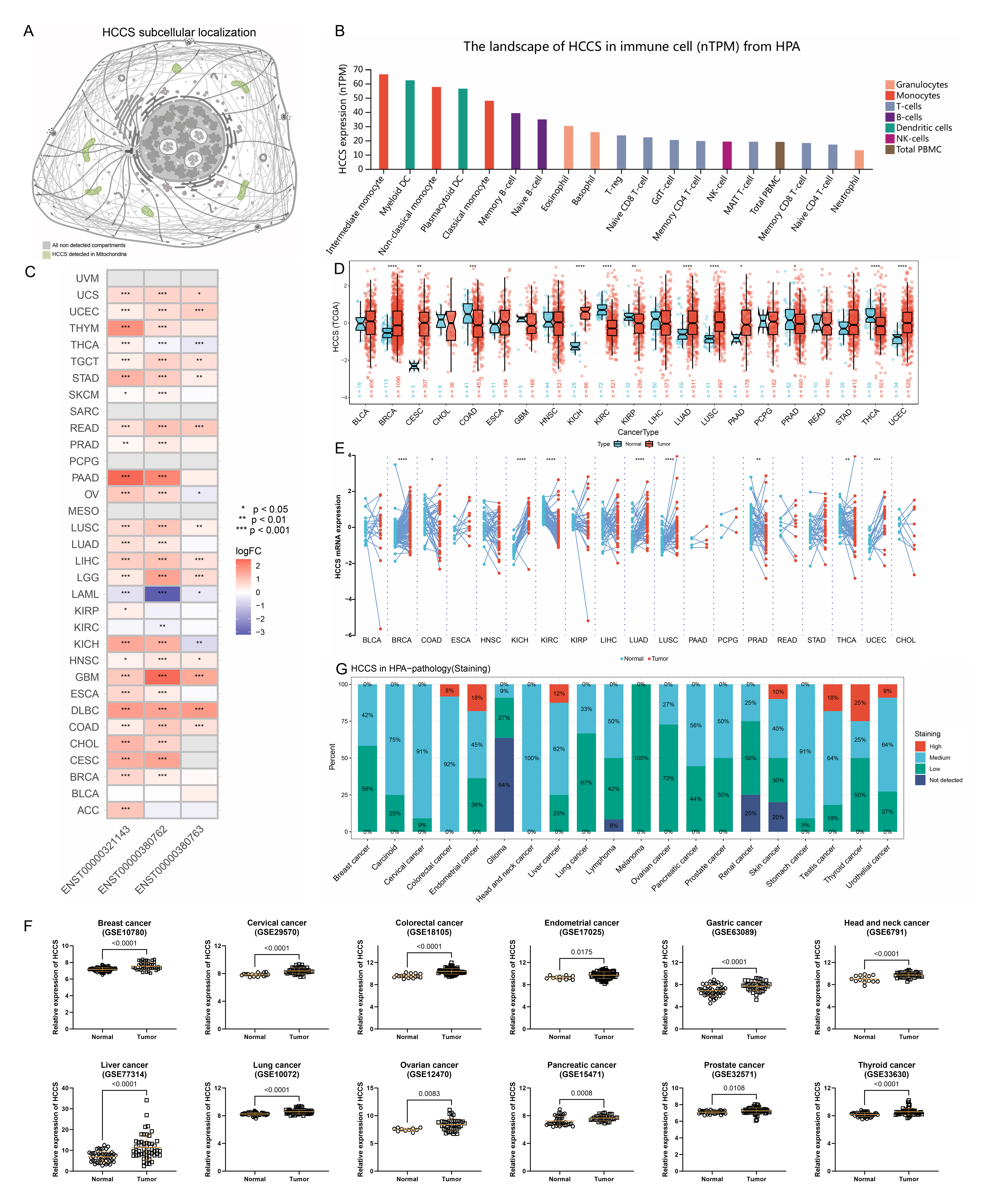


**Supplementary Figure 1.** (A) Subcellular localization analysis showed that HCCS was mainly localized in mitochondria; (B) The landscape of HCCS in immune cell from HPA; (C) Evaluate the differential expression levels of HCCS transcripts in pan-cancer normal and tumor samples; (D)Evaluation of HCCS differential expression levels in normal and tumor samples based on TCGA; (E) Evaluation of the differential expression levels of HCCS based on TCGA tumor samples and paired normal samples; (F) Data from multiple GEO datasets were used to validate the significant upregulation of HCCS in malignant tumors; (G) Statistical analysis of immunohistochemical staining results of HCCS gene in different types of tumors.

**

**

**Supplementary Figure 2.** (A) Classification of tissue regions into malignant (Mal) and non-malignant (nMal) based on the proportion of malignant cells in each microregion; (B) Single-cell expression profiling landscape of HCCs in pan-cancer.

**

**

**Supplementary Figure 3.** (A) Correlation analysis between HCCS copy number variation and mRNA expression in multiple tumor types; (B, C) Survival analysis revealed prognostic differences between HCCS mutants and wild-types; (D) Pan-cancer heatmap visualization of the average immune response and genomic status at different HCCS expression levels; (E, F) Survival analysis revealed prognostic differences between high and low methylation levels of HCCS; (G) Differences in PSI values ​​between normal and tumor tissues in Pan-Cancer (HCCS_AD_88468); (H-J) PSI differences when comparing tumors and corresponding healthy or adjacent tissues, and the association between HCCS_AD_88468 events and prognosis.


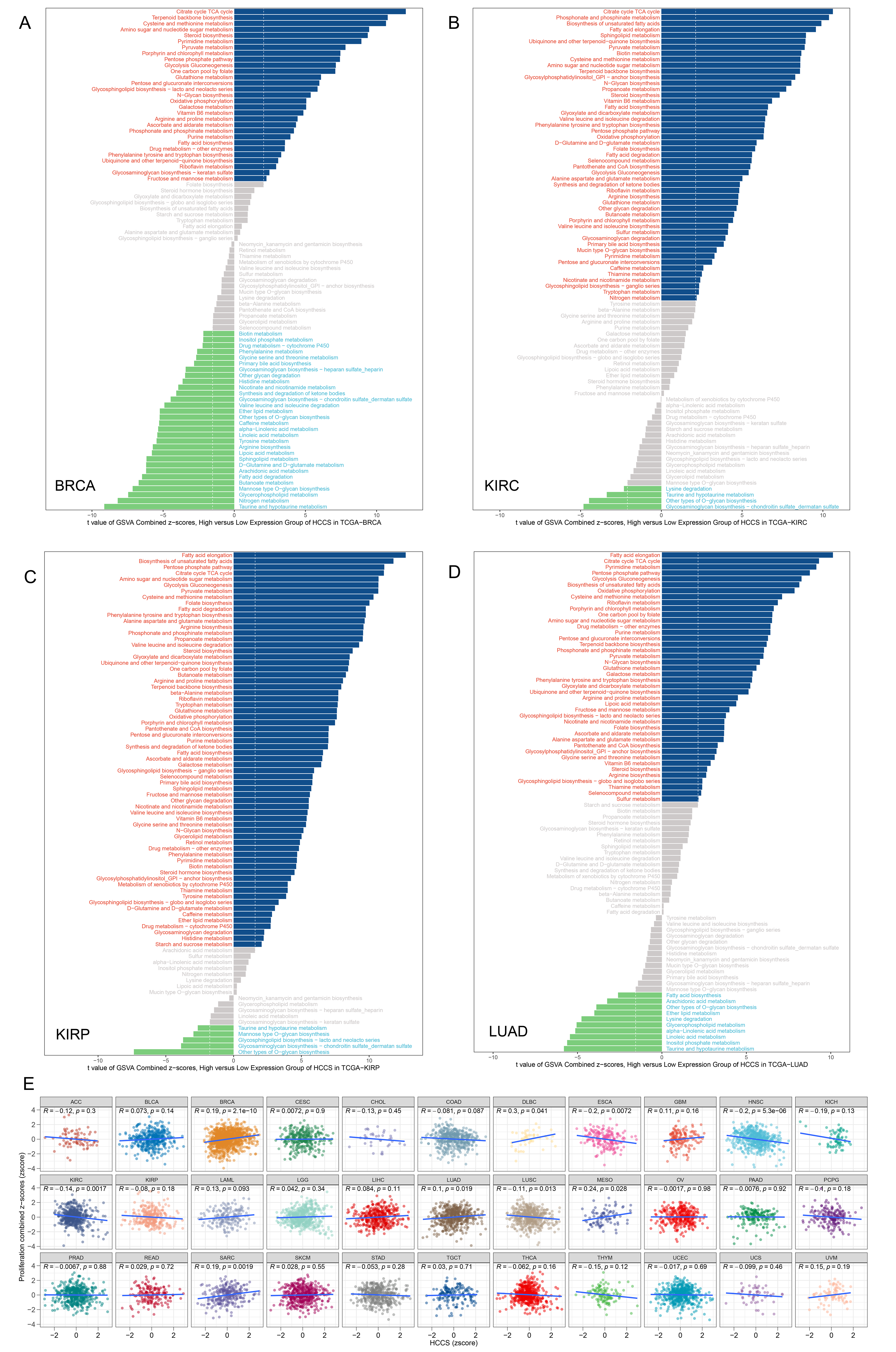


**Supplementary Figure 4.** (A-D) Differences in GSVA scores of metabolic pathways between high and low expression groups of HCCS genes in BRCA, KIRC, KIRP, and LUAD; (E) Correlation between HCCS expression and tumor proliferation activity. Scatter plots displaying Pearson correlation between HCCS levels and proliferation combined z-scores (based on CancerSEA) across TCGA. Consistently low correlation coefficients (R < 0.3) suggest that the biological impact of HCCS is distinct from canonical cell proliferation programs.





**Supplementary Figure 5.** (A) Spearman correlation coefficient between HCCS and pan-cancer immune signature; (B, C) Differential expression of HCCS high and low expression groups among pan-cancer immune subtypes; (D,E) HCCS was significantly correlated with step 1 (Release of cancer cell antigens) and step 5 (Infiltration of immune cells into tumors) of the pan-cancer tumor immunity cycle.





**Supplementary Figure 6.** (A, B) The ROC curve was used to evaluate the diagnostic efficacy of HCCS gene expression in distinguishing tumor group from normal group; (C) Kaplan-Meier survival analysis to evaluate the association between HCCS and tumor DFI, DSS, and PFI.

**

**

**Supplementary Figure 7.** (A-L) Validation of the prognostic value of HCCS in LGG, LIHC, and LUAD based on the GEO dataset.
